# Beta 2-microglobulin is an independent risk marker of acute kidney injury in adult patients with hemophagocytic lymphohistiocytosis

**DOI:** 10.1007/s40620-024-01949-0

**Published:** 2024-05-12

**Authors:** Mengya Zhao, Jingfeng Liu, Haizhou Zhuang, Yu Qiu, Zhanghuan He, Jin Lin, Meili Duan

**Affiliations:** Department of Critical Care Medicine, Beijing Friendship Hospital, Capital Medical University, 95 Yong-An Road, Xuan Wu District, Beijing, 100050 China

**Keywords:** β2-microglobulin, Acute kidney injury, Hemophagocytic lymphohistiocytosis

## Abstract

**Background and Aims:**

The role of beta2-microglobulin (β2-MG) in predicting acute kidney injury (AKI) in hemophagocytic lymphohistiocytosis patients has been poorly studied. This study aimed to analyze the clinical characteristics of hemophagocytic lymphohistiocytosis patients and identify risk factors that predict AKI development.

**Methods:**

This retrospective observational cohort study conducted at a single-center involved 938 patients diagnosed with hemophagocytic lymphohistiocytosis, who were divided into AKI  group and non-AKI group. Patient data were collected and analyzed using univariate and multivariate binary logistic regression to identify potiential risk factors associated with AKI occurrence.

**Results:**

Among the enrolled patients, 486 were male (51.9%), the median age was 37 years (interquartile range, 28.0, 52.0), 58.4% experienced AKI. Mechanical ventilation (8.0% vs. 0.8%) and vasopressor support (21.7% vs. 4.1%) occurred at significantly higher rates in the AKI group compared to the non-AKI group, with significantly higher in-hospital mortality (5.5% vs. 1.3%) and 28-day mortality (12.8% vs. 5.4%). When β2-MG was used as a continuous variable, multifactorial analysis showed that β2-MG, transplantation, and vasopressor support were independently associated with risk for the development of AKI.

**Conclusions:**

The incidence of morbidity and mortality in patients with hemophagocytic lymphohistiocytosis complicated by AKI remains high. Monitoring levels of β2-MG may provide clinicians with timely indicators of changes in renal function,  facilitating adjustments to treatment strategies.

**Graphical abstract:**

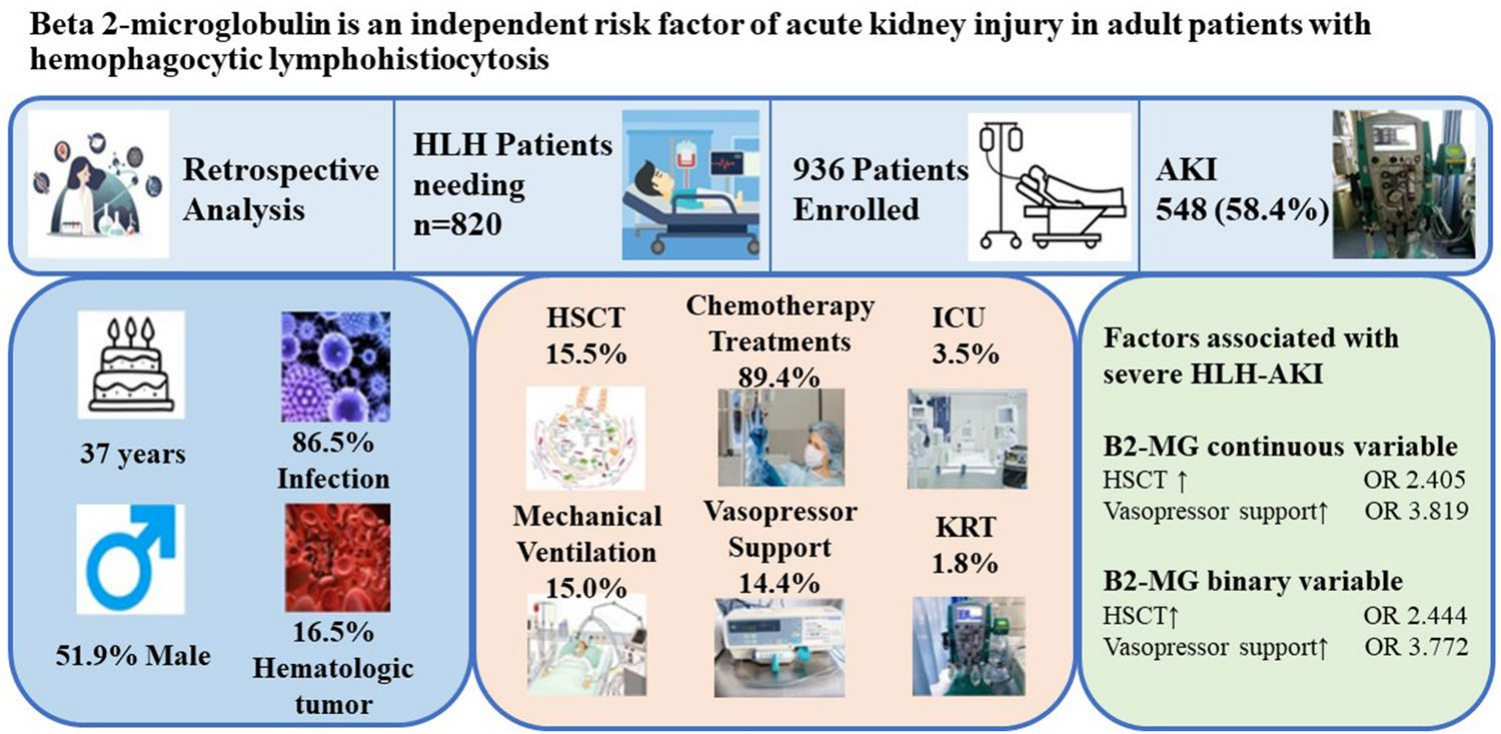

## Introduction

Hemophagocytic syndrome is a disease resulting from an excessive pathological inflammatory response caused by immune dysfunction, which can lead to cytokine storms and widespread organ dysfunction, including kidney injury. Renal involvement, especially acute kidney injury (AKI), is seen in up to 50% of patients with hemophagocytic syndrome [1], and current studies suggest that AKI is associated with poor patient prognosis [1–3]. Reports on AKI in hemophagocytic syndrome mainly include case studies or small case series, and the only two large studies on patients with hemophagocytic syndrome and AKI confim that AKI is a negative prognostic marker in these cases [1, 4].

The current diagnosis of AKI is mainly based on serum creatinine levels, according to the Kidney Disease Improving Global Outcomes (KDIGO) criteria published in 2012 [5]. However, because creatinine level is influenced by age, nutritional status, and muscle mass, it does not fully represent renal function [6]. Herrero-Morín JD [7] found that beta2-microglobulin (β2-MG) levels are different in patients with and without AKI.

β2-MG is an endogenous, low-molecular-weight serum protein secreted by lymphocytes and most other nucleated cells. Serum β2-MG is produced constantly and since the kidney is the only organ that excretes β2-MG, serum β2-MG, like serum creatinine, correlates with glomerular filtration rate (GFR). As such, β2-MG has been shown to predict AKI occurrence and indicate poor prognosis in populations with different diseases [8–13].

In this study, we describe the relationship between β2-MG levels and the occurrence of AKI in patients with hemophagocytic syndrome in a large series of 938 patients.

## Materials and methods

### Study population

We screened 1265 adult patients with a diagnosis of hemophagocytic syndrome admitted to Beijing Friendship Hospital, Capital Medical University, between 2014 and 2020 for inclusion in this study. Patients were excluded based on the following criteria: age < 18 years old, more than 1 year after diagnosis of hemophagocytic syndrome, missing data greater than 10%, patients who stopped treatment, continuous renal replacement therapy (CRRT) in the past month, lack of β2-MG measurement. Finally, 938 patients were included and divided into the AKI group (*n* = 548) and non-AKI group (*n* = 390).

### Data collection

Patient information was collected through the hospital's electronic medical records, and included general data, vital signs, laboratory data, medication records, etiology of hemophagocytic syndrome, treatments, length of stay, and prognosis. General data included sex, age, body mass index, comorbid conditions. Laboratory data included white blood cell count, hemoglobin, platelet count, C-reactive protein (CRP), creatinine, β2-MG, serum creatinine. Treatments mainly included chemotherapy, hematopoietic stem cell transplantation (HSCT), mechanical ventilation, use of vasopressor amines, intensive care unit (ICU) admission, and CRRT. The primary outcome was the occurrence of AKI, and the secondary outcome was death.

### Definitions

Hemophagocytic lymphohistiocytosis was diagnosed using the hemophagocytic syndrome-2004 criteria [14], and AKI was defined using the KDIGO-2012 standard [5].

All laboratory tests were collected at the time of hemophagocytic syndrome diagnosis. Requisites included availability of routine blood analyses carried out within one week from diagnosis, and organ function tests within two weeks before and after diagnosis. Baseline creatinine was defined as the creatinine value at the first visit, but if not recorded, we used the lowest creatinine value in the first week after admission as the baseline value.

### Statistical analysis

Data were analyzed using SPSS 25.0 (IBM Corp., USA). All continuous variables are expressed as mean ± standard deviation or the median and interquartile range (IQR), and categorical variables are presented as *n* (%). An independent sample *t*-test was applied for continuous variables conforming to the normal distribution, and the Mann–Whitney *U* test was used for continuous variables with non-normal distribution. The chi-square test was applied for categorical variables. Subgroup analysis was conducted according to the KDIGO classification of AKI. Univariate and multivariate binary logistic regression was applied for the analysis of risk factors for the occurrence of AKI. The odds ratio (OR) and 95% confidence interval (95%CI) were calculated. *P* < 0.05 was considered statistically significant.

The sample size was calculated based on the principle of 1 predictor matching 10 outcome events. The sample size was equal to the number of predictors*10/incidence of outcome. Forty-one variables were tested, and the sample size of 820 cases was calculated based on the 50% incidence of AKI reported in previous studies.

## Results

### Study population and characteristics

**Fig. 1:**
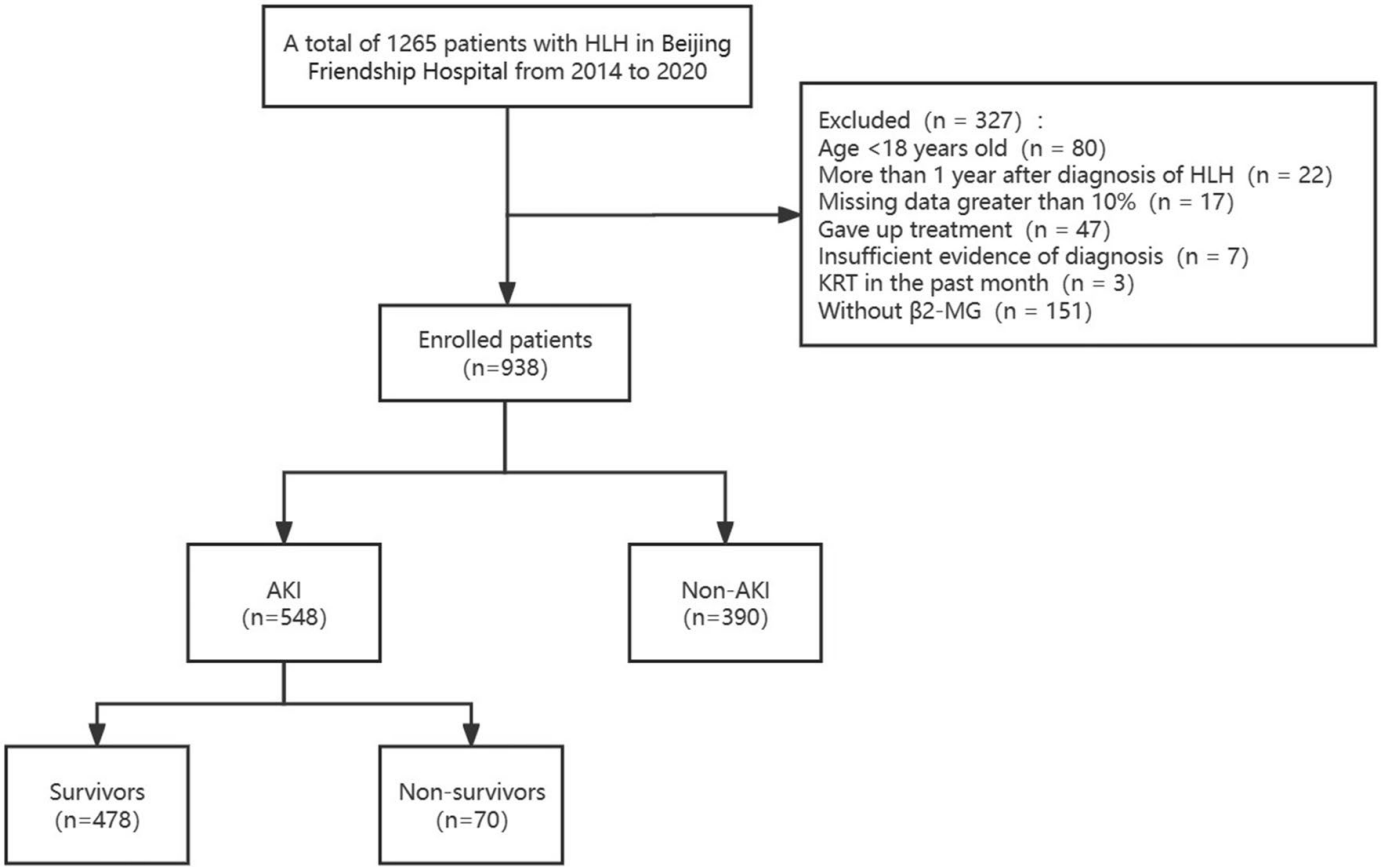
Flow chart for patient enrollment

The study flow chart is presented in Fig. 1. The baseline characteristics of the patients are shown in Table 1, including demographics, laboratory data, etiology of hemophagocytic syndrome, and comorbid conditions. The primary treatment modalities and associated prognostic factors are detailed in Table 2.

**Table 1:** Baseline characteristics in all patients and comparison between AKI and non‐AKI patients

Characteristic	All (*n* = 938)	AKI group (*n* = 548)	Non-AKI group (*n* = 390)	*P* value
Age (years), median (IQR)	37.0 (28.0, 52.0)	36.0 (28.0, 52.0)	40.0 (27.0, 52.0)	0.192
Male sex, n (%)	486 (51.9)	309 (56.4)	177 (45.4)	0.001
BMI (kg/m^2^), median (IQR)	21.7 (19.5, 24.0)	21.6 (19.5, 23.7)	21.8 (19.6, 24.2)	0.061
Infection at hospital admission, n (%)	811 (86.5)	496 (90.5)	315 (80.8)	< 0.001
Hematologic tumor, n (%)	155 (16.5)	106 (19.3)	49 (12.6)	0.006
Causes of Disease, n (%)				
Single Etiology (There’s only one trigger for hemophagocytic lymphohistiocytosis)	155 (16.5)	105 (19.2)	50 (12.8)	< 0.001
Infection	451 (48.1)	290 (52.9)	161 (41.3)	< 0.001
Tumor	363 (38.7)	235 (42.9)	128 (32.8)	0.002
Immune	113 (12.0)	51 (9.3)	62 (15.9)	0.002
Genetic	23 (2.5)	12 (2.2)	11 (2.8)	0.538
Comorbid condition, n (%)				
Single Combination (At the time of inclusion, the patients were comorbid with only one underlying disease)	734 (78.3)	438 (79.9)	296 (75.9)	0.140
Hypertension	79 (8.4)	39 (7.1)	40 (10.3)	0.088
Diabetes	69 (7.4)	40 (7.3)	29 (7.4)	0.937
Cardiac disease	28 (3.0)	13 (2.4)	15 (3.8)	0.191
Chronic liver disease	84 (9.0)	46 (8.4)	38 (9.7)	0.476
Chronic kidney disease	12 (1.3)	7 (1.3)	5 (1.3)	0.995
Chronic lung disease	63 (6.7)	41 (7.5)	22 (5.6)	0.267
Immune disease	59 (6.3)	33 (6.0)	26 (6.7)	0.689
Ulcer of digestive tract	5 (0.5)	3 (0.5)	2 (0.5)	0.943
Cerebrovascular disease	19 (2.0)	12 (2.2)	7 (1.8)	0.672
Peripheral vascular disease	5 (0.5)	3 (0.5)	2 (0.5)	0.943
Non-hematologic tumor	34 (3.6)	17 (3.1)	17 (4.4)	0.310
Biochemical data, Mean (IQR)				
WBC count (10^9^/L)	2.7 (1.6, 5.3)	2.6 (1.6, 5.2)	2.9 (1.6, 5.3)	0.360
Platelet count (10^9^/L)	67.0 (34.0, 118.3)	64.0 (33.0, 109.0)	74.0 (37.0, 134.8)	0.010
Hemoglobin (g/L)	93.0 (77.0, 110.0)	92.0 (77.0, 108.3)	94.0 (78.0, 112.0)	0.173
CRP (mg/L)	19.3 (4.1, 59.9)	23.0 (6.4, 67.0)	12.0 (2.9, 50.8)	< 0.001
Total bilirubin (umol/L)	22.2 (13.7, 45.5)	25.7 (15.0, 56.3)	18.9 (12.3, 34.2)	< 0.001
ALT (U/L)	71.0 (34.0, 163.3)	74.5 (37.0, 161.8)	66.5 (31.2, 164.5)	0.762
AST (U/L)	78.0 (35.0, 182.8)	84.0 (38.2, 194.0)	68.4 (32.6, 165.1)	0.083
AST/ALT	1.1 (0.7, 1.8)	1.2 (0.8, 1.9)	1.0 (0.6, 1.7)	< 0.001
LDH (U/L)	494.0 (312.0, 909.0)	535.6 (337.0, 1015.5)	448.5 (273.8, 776.5)	< 0.001
Albumin (g/L)	30.5 (26.6, 35.1)	29.3 (25.9, 33.7)	32.4 (27.7, 36.5)	< 0.001
Creatinine (umol/L)	53.6 (44.1, 65.8)	55.6 (44.8, 70.4)	51.6 (43.4, 61.5)	< 0.001
Triglycerides (mmol/L)	2.3 (1.6, 3.4)	2.4 (1.6, 3.6)	2.2 (1.6, 3.1)	0.027
β2-MG (mg/L)	3.7 (2.5, 5.4)	4.2 (2.8, 6.2)	3.2 (2.1, 4.5)	< 0.001
Serum ferritin (ng/ml)	2024.1 (1130.8, 7111.2)	2146.6 (1184.5, 8142.1)	2000.0 (999.9, 6300.6)	0.054
Fibrinogen (g/L)	1.8 (1.2, 2.9)	1.8 (1.1, 2.8)	1.9 (1.3, 3.0)	0.017
sCD25 (ng/ml)	18,925.5 (7220.9, 39,063.2)	24,314.0 (8265.5, 42,285.5)	12,441.0 (4673.0, 30,075.7)	< 0.001
NK cell activity (%)	14.5 (12.7, 16.5)	14.5 (12.7, 16.7)	14.6 (12.7, 16.3)	0.604
SOFA score, median (IQR)	3.0 (2.0, 5.0)	3.0 (2.0, 5.0)	3.0 (2.0, 5.0)	0.452

*HLH* hemophagocytic lymphohistiocytosis, *AKI* acute kidney injury, *IQR* interquartile range; *BMI* body mass index, *WBC* white blood cell, *CRP* C-reactive protein, *ALT* alanine aminotransferase, *AST* aspartate aminotransferase, *LDH* lactate dehydrogenase, *β2-MG* beta2-microglobulin, *sCD25* soluble CD25, *NK* natural killer, *SOFA* sequential organ failure assessment

**Table 2:** Patient treatments and outcomes

Characteristic	All (*n* = 938)	AKI group (*n* = 548)	Non-AKI group (*n* = 390)	*P* value
Hematopoietic stem cell transplantation* n* (%)	145 (15.5)	121 (22.1)	24 (6.2)	< 0.001
Chemotherapy treatments,* n* (%)	839 (89.4)	524 (95.6)	315 (80.8)	< 0.001
DEP,* n* (%)	513 (61.1)	350 (63.9)	163 (41.8)	
HLH94,* n* (%)	109 (13.0)	52 (9.5)	57 (14.6)	
HLH2004, * n* (%)	43 (5.1)	31 (5.7)	12 (3.1)	
Others, * n* (%)	174 (20.7)	91 (17.4)	83 (26.3)	
ICU admission, * n* (%)	33 (3.5)	33 (6.0)	0 (0)	< 0.001
Invasive mechanical ventilation, * n* (%)	47 (5.0)	44 (8.0)	3 (0.8)	< 0.001
Vasopressor support, * n* (%)	135 (14.4)	119 (21.7)	16 (4.1)	< 0.001
CRRT, * n* (%)	17 (1.8)	17 (3.1)	0 (0)	< 0.001
Hospital length of stay (days)	12 (8, 18)	13 (9, 22)	10 (7, 14)	< 0.001
In-hospital Mortality, * n* (%)	35 (3.7)	30 (5.5)	5 (1.3)	0.001
28-day mortality, * n* (%)	91 (9.7)	70 (12.8)	21 (5.4)	< 0.001

*HSCT* hematopoietic stem cell transplantation, *ICU* intensive care unit, *CRRT* continuous renal replacement therapy

A total of 938 patients were included in this study, including 309 males (56.4%) in the AKI group and 177 males (45.4%) in the non-AKI group. The median age of patients in both groups was 36.0 (28.0, 52.0) years and 40.0 (27.0, 52.0) years, respectively. In our study, infection was more common than hematologic tumor, accounting for 451 cases (48.1%) of infection and 363 cases (38.7%) of tumor, with a higher percentage of patients with hemophagocytic syndrome due to infection and tumor (52.9% and 42.9%) developing AKI. Among these patients, there were 155 patients (16.5%) with a single etiology of hemophagocytic syndrome and 125 patients (13.3%) with no identified cause. The probability of developing AKI was higher in patients with multiple etiology of hemophagocytic syndrome.

### Risk factors for the occurrence of AKI and β2-MG as a risk marker of AKI

The baseline creatinine levels were 55.6 (44.8, 70.4) µmol/L and 51.6 (43.4, 61.5) µmol/L in the AKI and non-AKI groups, respectively, with significantly higher baseline creatinine levels in the AKI group (*P* < 0.001). The number of cases of AKI that occurred within one year after diagnosis of hemophagocytic syndrome was 548 (58.4%), including 292 (53.3%) patients with KDIGO grade 1 AKI, 130 (23.7%) patients with KDIGO grade 2 AKI, and 126 (23.0%) patients with KDIGO grade 3 AKI. Acute kidney injury occurred more frequently in men (56.4%), and there was no significant relationship between age and AKI. Regarding laboratory data, patients in the AKI group had lower levels of platelets, fibrinogen, and serum albumin and higher levels of total bilirubin, lactate dehydrogenase, serum creatinine, triglycerides, soluble CD25 (sCD25), β2-MG, and CRP. There were no significant differences in the sequential organ failure assessment scores of patients in the two groups (Table 1).

In terms of clinical treatment, patients in the AKI group had a higher prevalence of chemotherapy treatments (95.6% vs. 80.8%, *P* < 0.001) and HSCT (22.1% vs. 6.2%, *P* < 0.001), required more advanced organ support therapy, had a significantly higher need for invasive mechanical ventilation (8.0% vs. 0.8%), and vasopressor need (21.7% vs. 4.1%). Of note, there are three main treatment regimens for hemophagocytic syndrome, namely DEP (etoposide, steroids and liposomal doxorubicin), HLH94 (steroids and etoposide) and HLH2004 (steroids, etoposide and cyclosporine). The DEP regimen was associated with the highest rate of AKI (63.9%) (Table 3). Six percent of patients in the AKI group were admitted to the ICU, while none in the non-AKI group were admitted to the ICU. The hospital length of stay was significantly longer in the AKI group (13 d vs. 10 d, *P* < 0.001). In-hospital mortality and 28-day mortality were higher in the AKI group compared to the non-AKI group (5.5% vs. 1.3% and 12.8% vs. 5.4%) and the difference is statistically significant (Table 2).

**Table 3:** Comparison of the effect of chemotherapy regimens on AKI between groups

Chemotherapy regimens	DEP vs HLH94	DEP vs. HLH2004	DEP vs. Others	HLH94 vs. HLH2004	HLH94 vs. Others	HLH2004 vs Others
P value	0.0003*	0.999	0.0004*	0.028*	0.999	0.068

*DEP* etoposide, steroids and liposomal doxorubicin, *HLH94* steroids and etoposide, *HLH2004* steroids, etoposide and cyclosporine; *p < 0.05

**Fig. 2:**
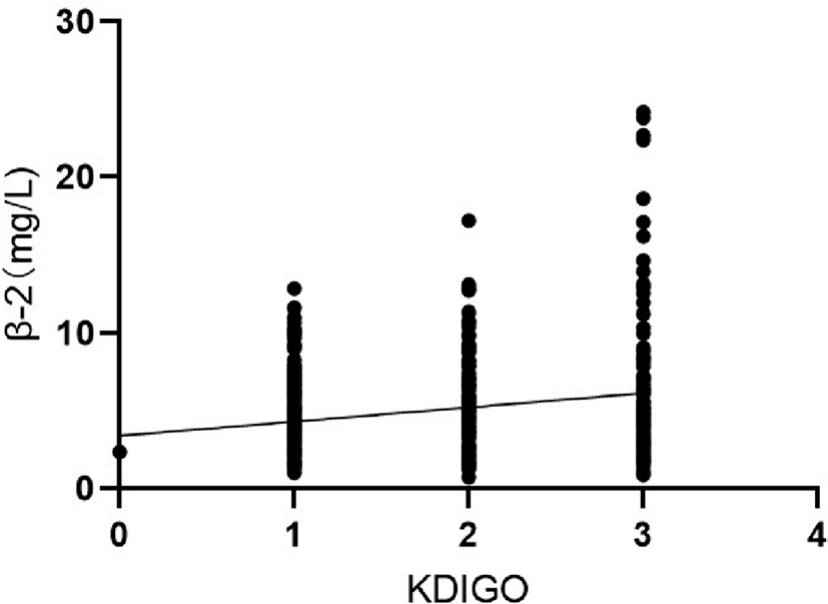
Prevalence of AKI in patients grouped according to β2-MG level

Multifactorial binary regression analysis revealed that the presence of infection upon admission (*P* = 0.016, OR = 1.837, 95%CI: 1.121–3.012), CRP (*P* = 0.001, OR = 1.006, 95%CI: 1.002–1.010), β2-MG (*P* < 0.001, OR = 1.239, 95%CI: 1.145–1.340), chemotherapy (*P* < 0.001, OR = 4.599, 95%CI: 2.548–8.298), HSCT (*P* < 0.001, OR = 3.361, 95%CI: 1.964–5.752), and use of vasopressors (*P* < 0.001, OR = 4.837, 95%CI: 2.531–9.244) were independently associated with the development of AKI (Table 4). When β2-MG was used as a categorical variable in the multifactor binary regression analysis, creatinine level (*P* = 0.004, OR = 1.012, 95%CI: 1.004–1.021) was an independent risk factor for the development of AKI in addition to the above indicators, while fibrinogen (*P* = 0.025, OR = 0.861, 95%CI: 0.756–0.982) was a protective factor for the development of AKI (Table 5). The risk of AKI increased with increasing levels of β2-MG (Fig. 2).

**Table 4:** Univariate and multivariate logistic regression analysis for risk factors of AKI using β2-MG as a continuous variable

Variables	Unit	Univariate model		Multivariate model	
		*P* value	OR (95%CI)	*P* value	OR (95%CI)
Infection at hospital admission no./total no. (%)	Yes or No	< 0.001	2.271 (1.552–3.324)	0.016	1.837 (1.121–3.012)
CRP (mg/L)	Per 1 mg/l increment	0.001	1.005 (1.002–1.008)	0.001	1.006 (1.002–1.010)
β2-MG (mg/L)	Per 1 mg/l increment	< 0.001	1.270 (1.190–1.355)	< 0.001	1.239 (1.145–1.340)
HSCT	Yes or No	< 0.001	4.321 (2.729–6.843)	< 0.001	3.361 (1.964–5.752)
Chemotherapy	Yes or No	< 0.001	5.198 (3.215–8.405)	< 0.001	4.599 (2.548–8.298)
Vasopressor support	Yes or No	< 0.001	6.484 (3.779–11.127)	< 0.001	4.837 (2.531–9.244)

*AKI* acute kidney injury, *β2-MG* beta2-microglobulin, *CRP* C-reactive protein, *HSCT* hematopoietic stem cell transplantation, *OR* odds ratio, *CI* confidence interval

**Table 5:** Univariate and multivariate logistic regression analysis for risk factors of AKI using β2-MG as a binary variable

Variables	Unit	Univariate model		Multivariate model	
		*P* value	OR (95%CI)	*P* value	OR (95%CI)
Infection at hospital admission no./total no. (%)	Yes or No	< 0.001	2.271 (1.552–3.324)	0.010	1.921 (1.165–3.168)
CRP (mg/L)	Per 1 mg/l increment	0.001	1.005 (1.002–1.008)	< 0.001	1.008 (1.004–1.012)
β2-MG	Yes or No	< 0.001	2.255 (1.681–3.025)	< 0.001	1.866 (1.324–2.629)
HSCT	Yes or No	< 0.001	4.321 (2.729–6.843)	< 0.001	3.359 (1.962–5.751)
Chemotherapy	Yes or No	< 0.001	5.198 (3.215–8.405)	< 0.001	4.955 (2.729–8.997)
Vasopressor support	Yes or No	< 0.001	6.484 (3.779–11.127)	< 0.001	4.971 (2.603–9.493)
Fibrinogen (g/L)	Per 1 g/l increment	0.035	0.904 (0.824–0.993)	0.025	0.861 (0.756–0.982)
Creatinine (umol/L)	Per 1 umol/l increment	< 0.001	1.013 (1.007–1.019)	0.004	1.012 (1.004–1.021)

*AKI* acute kidney injury, *β2-MG* beta2-microglobulin, *CRP* C-reactive protein, *HSCT* hematopoietic stem cell transplantation, *OR* odds ratio, *CI* confidence interval

### Risk factors for the occurrence of severe AKI

In the subgroup analysis of AKI patients, a higher proportion of men developed severe AKI, with 63.1% and 65.1% of patients having KDIGO grades 2 and 3 AKI, respectively. When HLH was caused by immune factors, KDIGO grade 1 AKI predominated. Total bilirubin, serum creatinine, β2-MG, and sCD25 levels were increased and albumin levels were decreased in patients with severe AKI, and all were statistically significant (Table 6). The higher the KDIGO grade, the higher the proportion of patients needing ICU admission, receiving HSCT, mechanical ventilation, vasopressor support, and CRRT. The main drugs potentially involved are reported in Table 7.

**Table 6:** Baseline characteristics in all AKI patients with HLH compared between different KDIGO grades

Characteristic	AKI group (*n* = 548)	KDIGO 1 (*n* = 292)	KDIGO 2 (*n* = 30)	KDIGO 3 (*n* = 126)	*P* value
Male sex,* n* (%)	309 (56.4)	145 (49.7)	82 (63.1)	82 (65.1)	0.003
Causes of Disease, * n* (%)					
Immune	51 (9.3)	39 (13.4)	7 (5.4)	5 (4.0)	0.002
Comorbid condition, * n* (%)					
Cerebrovascular disease	12 (2.2)	4 (1.4)	7 (5.4)	1 (0.8)	0.016
Biochemical data, Mean (IQR)					
Total bilirubin (umol/L)	25.7 (15.0, 56.3)	23.1 (13.4, 44.1)	28.1 (15.8, 62.6)	32.2 (18.3, 76.1)	0.002
Albumin (g/L)	29.3 (25.9, 33.7)	30.5 (26.2, 34.5)	28.9 (25.4, 33.1)	28.2 (26.0, 32.0)	0.006
Creatinine (umol/L)	55.6 (44.8, 70.4)	53.2 (44.7, 63.6)	62.0 (46.6, 82.6)	57.7 (44.5, 98.9)	< 0.001
β2-MG (mg/L)	4.2 (2.8, 6.2)	3.9 (2.6, 5.7)	4.6 (2.8, 6.9)	4.5 (3.1, 7.1)	< 0.001
sCD25 (ng/ml)	24,314.0 (8265.5, 42,285.5)	20,356.4 (7500.0, 39,536.5)	26,320.5 (9241.5, 44,000)	31,052.0 (13,278.0, 44,000)	0.005

*HLH* hemophagocytic lymphohistiocytosis, *AKI* acute kidney injury, *IQR* interquartile range, *β2-MG* beita2-microglobulin, *sCD25* soluble CD25

**Table 7:** Patient treatments and outcomes

Characteristic	AKI group (*n* = 548)	KDIGO 1 (*n* = 292)	KDIGO 2 (*n* = 130)	KDIGO 3 (*n* = 126)	*P* value
HSCT, * n* (%)	121 (22.1)	49 (16.8)	33 (25.4)	39 (31.0)	0.003
Chemotherapy, * n* (%)	524 (95.6)	279 (95.5)	125 (96.2)	120 (95.2)	0.934
Nephrotoxic drugs before AKI, * n* (%)	247 (75.5)	137 (75.3)	56 (71.8)	54 (80.6)	0.204
Diuretics, * n* (%)	117 (21.4)	52 (17.8)	26 (20.0)	39 (31.0)	0.010
ICU admission, * n* (%)	33 (6.0)	5 (1.7)	11 (8.5)	17 (13.5)	< 0.001
Invasive mechanical ventilation, * n* (%)	44 (8.0)	11 (3.8)	14 (10.8)	19 (15.2)	< 0.001
Vasopressor support, * n* (%)	119 (21.7)	31 (10.6)	27 (20.8)	61 (48.4)	< 0.001
CRRT, * n* (%)	17 (3.1)	1 (0.3)	4 (3.1)	12 (9.5)	< 0.001
Hospital length of stay (days), Mean (IQR)	13 (9, 22)	13 (9, 20)	13 (8, 22)	14 (8, 25)	0.124
In-hospital mortality in-hospital, * n* (%)	30 (5.5)	4 (1.4)	12 (9.2)	14 (11.1)	< 0.001
28-day mortality at 28 days, * n* (%)	70 (12.8)	22 (7.5)	21 (16.2)	27 (21.4)	< 0.001

*AKI* acute kidney injury, *HSCT* hematopoietic stem cell transplantation, *IQR* interquartile range, *ICU* intensive care unit, *CRRT* continuous renal replacement therapy

Whether β2-MG is a continuous or categorical variable, multifactorial binary regression analysis for patients with severe AKI revealed that β2-MG, creatinine levels, HSCT, and vasopressor support were independent risk factors for the development of severe AKI (Tables 8, 9). However, when β-2MG was the categorical variable, bilirubin level was also an independent risk factor for severe AKI (Table 9).

**Table 8:** Univariate and multivariate logistic regression analysis for risk factors of severe AKI when β2-MG as a continuous variable

Variables	Unit	Univariate model		Multivariate model	
		*P* value	OR (95%CI)	*P* value	OR (95%CI)
β2-MG (mg/L)	Per 1 mg/l increment	< 0.001	1.164 (1.094–1.240)	0.018	1.096 (1.016–1.182)
Creatinine (umol/L)	Per 1 umol/l increment	< 0.001	1.022 (1.014–1.029)	< 0.001	1.022 (1.013–1.031)
HSCT	Yes or No	0.002	1.941 (1.287–2.925)	< 0.001	2.405 (1.528–3.783)
Vasopressor support	Yes or No	< 0.001	4.410 (2.804–6.937)	< 0.001	3.819 (2.324–6.276)

*AKI* acute kidney injury, *β2-MG* beta2-microglobulin, *HSCT* hematopoietic stem cell transplantation, *OR* odds ratio, *CI* confidence interval

**Table 9:** Univariate and multivariate logistic regression analysis for risk factors of severe AKI using β2-MG as a binary variable

Variables	Unit	Univariate model		Multivariate model	
		*P* value	OR (95%CI)	*P* value	OR (95%CI)
β2-MG (mg/L)	Per 1 mg/l increment	0.001	1.810 (1.286–2.547)	0.047	1.487(1.005–2.200)
Creatinine (umol/L)	Per 1 umol/l increment	< 0.001	1.022 (1.014–1.029)	< 0.001	1.024 (1.015–1.033)
HSCT	Yes or No	0.002	1.941 (1.287–2.925)	< 0.001	2.444 (1.553–3.847)
Total bilirubin (umol/L)	Per 1 umol/l increment	0.013	1.002 (1.001–1.004)	0.035	1.002 (1.000–1.005)
Vasopressor support	Yes or No	< 0.001	4.410 (2.804–6.937)	< 0.001	3.772 (2.290–6.214)

*AKI* acute kidney injury, *β2-MG* beta2-microglobulin, *CRRT* continuous renal replacement therapy, *HSCT* hematopoietic stem cell transplantation, *OR* odds ratio, *CI* confidence interval

Among our patients, those with higher β2-MG levels made up a greater proportion of subjects receiving chemotherapy (*P* = 0.001), with longer hospital stays (*P* = 0.001), and no statistically significant in-hospital mortality (p = 0.358), but higher 28-day mortality (*P* = 0.020). β2-MG levels did not show a statistically significant effect on whether or not they received a transplant (*P* = 0.237). (Table 10).

**Table 10:** The relationship between β2-MG and patient treatments and outcomes

Characteristic	All (*n* = 938)	Increased β2-MG (*n* = 687)	Non-Increased β2-MG (*n* = 251)	*P* value
HSCT, * n* (%)	145 (15.5)	112 (16.3)	33 (13.1)	0.237
Chemotherapy treatments, * n* (%)	839 (89.4)	628 (91.4)	59 (23.5)	0.001*
Hospital length of stay (days), Mean (IQR)	12 (8, 18)	12 (8, 20)	11 (7, 16)	0.001*
In-hospital mortality, * n* (%)	35 (3.7)	28 (4.1)	7 (2.8)	0.358
28-day mortality, * n* (%)	91 (9.7)	76 (11.1)	15 (6.0)	0.020*

*β2-MG* beta2-microglobulin, *HSCT* hematopoietic stem cell transplantation, *IQR* interquartile range

*P < 0.05

## Discussion

This is the largest known case study of AKI in adults with hemophagocytic syndrome to date. The incidence of AKI in patients with hemophagocytic syndrome was 58.4% in this study, at difference with previous studies, likely due to variations in cases and populations. The proportion of hematologic malignancies was high in the studies by Aulagnon et al. and by Wang et al. (77% and 52.7%). The higher proportion of patients with infections (48.1%) and with malignancies (38.7%) in our study could be one of the reasons for the difference in incidence of AKI. AKI is seen in approximately two thirds of patients with septic shock [2, 15]. Previous studies have shown that a cytokine storm, which can induce secondary organ function impairment, is a common hallmark of both hemophagocytic syndrome and severe infections. Chemotherapy (95.6%) and bone marrow transplantation (22.1%) were represented in the AKI group. In this group, drug toxicity, primary disease, and tumor lysis after chemotherapy may significantly contribute to the development of AKI. Still, neither tumor nor underlying comorbidities showed significant correlation with AKI in our study. In addition, the patients in our study were from various geographic regions of China, and some patients had been previously evaluated and treated at their local medical facilities. This did not allow us to control for time to treatment and diagnosis in our study population.

β2-MG levels in our study were statistically different between the AKI and non-AKI groups and among KDIGO classification groups. β2-MG was shown to be independently associated with risk for the development of AKI in our regression analysis, suggesting β2-MG may play a role in the development of severe AKI, which is similar to what has been reported in studies involving pediatric patients [7], AKI with cerebral hemorrhage [9], and AKI after autologous stem cell transplantation in patients with multiple myeloma [12].

The mortality of patients with AKI remains high [16]. Hemophagocytic lymphohistiocytosis is associated with a high mortality rate, and the combination of AKI increases mortality. Creatinine levels do not fully reflect renal function in all patients, therefore, we need a more sensitive indicator than creatinine to reflect renal function. As a small protein that is easily filtered and is excreted only by the kidneys, β2-MG may be a marker of renal function [17–19].

Given the retrospective nature of this study, there were no data of the patients’ urine samples. Overall, our results suggest that β2-MG could support prediction of AKI and severe AKI. Patients with severe renal impairment had higher β2-MG levels, which may be related to the fact that a higher proportion of them received chemotherapy, and to the reduced renal excretion. Severe renal impairment is associated with higher mortality, which is in keeping with the findings of Astor et al. in kidney transplant patients [8].

To our knowledge, this is the first study to explore the association between serum β2-MG and the risk of developing AKI in patients with hemophagocytic syndrome. This study included the largest number of hemophagocytic syndrome patients with AKI. However, there are some limitations. First, this was a single-center retrospective study with limited strength in diagnostic evaluation. Second, the study lacked urine analysis and complete data. Third, we did not track the specific time of β2-MG measurement to understand the temporal cut-off point.

In conclusion, patients with hemophagocytic syndrome have a high risk of developing AKI, and the presence of AKI is associated with a poor prognosis. B2-MG is an independent marker of AKI and could be used to help clinicians identify high-risk patients earlier and take preventive or therapeutic measures to improve prognosis.

## Data Availability

The data that support the findings of this study are available from the corresponding author upon reasonable request.
